# Access to iodized salt in 11 low- and lower-middle-income countries: 2000 and 2010

**DOI:** 10.2471/BLT.15.160036

**Published:** 2015-12-01

**Authors:** Thach Duc Tran, Basil Hetzel, Jane Fisher

**Affiliations:** a Jean Hailes Research Unit, School of Public Health and Preventive Medicine, Monash University, Melbourne, Victoria 3004, Australia.; b International Council for Control of Iodine Deficiency Disorders, Women's and Children's Hospital, North Adelaide, Australia.

## Abstract

**Objective:**

To describe changes in household access to iodized salt in relation to socioeconomic factors.

**Methods:**

We extracted data on iodized household salt from Multiple Indicator Cluster Surveys conducted in 2000 and 2010. As part of the surveys, household salt samples were tested for iodization by standardized rapid-test kits that yield results to indicate whether salt is not iodized, inadequately iodized, (less than 15 parts per million, ppm), or adequately iodized (more than 15 ppm). We calculated indices of household salt iodization in 2000 and 2010, taking into account survey sampling weights. We explored associations between these indices and socioeconomic variables, both within and between countries.

**Findings:**

We analysed data from 105 162 households in 2000 and 144 018 households in 2010. Between 2000 and 2010, household coverage of adequately iodized salt increased by 6.1% (from 46.3% to 52.4%) on average, but with regional differences: coverage fell by 13.0% (from 77.5% to 64.5%) in the Central African Republic but improved by 40.4% (from 22.2% to 62.6%) in Sierra Leone. Improvements in coverage were higher in rural areas and among the poorest households, but within-country socioeconomic disparities remained. There were weak associations between changes in salt iodization and national level socioeconomic indicators.

**Conclusion:**

Overall, the coverage of adequately iodized household salt increased over the last decade. However, the changes varied widely among countries. The goal of universal salt iodization is still distant for many countries and requires renewed efforts by governments, bilateral and multilateral agencies and civil society.

## Résumé

**Objectif:**

Décrire les changements survenus dans l'accès des ménages à du sel iodé par rapport à divers facteurs socioéconomiques.

**Méthodes:**

Nous avons extrait des données sur le sel de cuisine iodé d'enquêtes par grappes à indicateurs multiples menées en 2000 et 2010. Dans le cadre de ces enquêtes, l'iodation d'échantillons de sel de cuisine a été évaluée à l'aide de kits de tests rapides standardisés qui ont permis d'obtenir des résultats indiquant que le sel n'est pas iodé, insuffisamment iodé (moins de 15 parties par million, ppm) ou suffisamment iodé (plus de 15 ppm). Nous avons calculé les indices d'iodation du sel de cuisine en 2000 et 2010 en tenant compte des poids d'échantillonnage des enquêtes. Nous avons examiné les liens entre ces indices et plusieurs variables socioéconomiques dans et entre les pays.

**Résultats:**

Nous avons analysé des données provenant de 105 162 ménages en 2000 et 144 018 ménages en 2010. Entre 2000 et 2010, le nombre de ménages consommant du sel suffisamment iodé a augmenté de 6,1% (de 46,3% à 52,4%) en moyenne, mais des différences régionales ont été constatées: ce taux a baissé de 13,0% (de 77,5% à 64,5%) en République centrafricaine, mais a augmenté de 40,4% (de 22,2% à 62,6%) en Sierra Leone. Il a été noté que les améliorations étaient plus importantes dans les zones rurales et au sein des ménages les plus pauvres, mais que des disparités socioéconomiques subsistaient au sein des pays. Les liens entre les changements dans l'iodation du sel et les indicateurs socioéconomiques au niveau national se sont révélés faibles.

**Conclusion:**

Dans l'ensemble, l'accès à du sel de cuisine suffisamment iodé s'est amélioré au cours de la dernière décennie. Il a cependant été observé que les changements variaient considérablement d'un pays à l'autre. Dans de nombreux pays, l'objectif de l'iodation universelle du sel est loin d'être atteint et exige des gouvernements, des organismes bilatéraux et multilatéraux et de la société civile qu'ils renouvellent leurs efforts.

## Resumen

**Objetivo:**

Describir los cambios del acceso doméstico a sal yodada en relación con factores socioeconómicos.

**Métodos:**

Se extrajeron datos sobre la sal doméstica yodada de encuestas de indicadores múltiples a nivel de grupos realizadas en 2000 y 2010. Como parte de las encuestas, se analizaron las muestras de sal doméstica para comprobar su yodación mediante kits de pruebas rápidas estandarizadas, cuyos resultados indicaron si la sal no estaba yodada, estaba insuficientemente yodada, menos de 15 partes por millón (ppm) o suficientemente yodada (más de 15 ppm). Se calcularon los índices de yodación de la sal doméstica en el 2000 y el 2010, teniendo en cuenta las ponderaciones de muestreo de las encuestas. Se exploraron las relaciones entre dichos índices y las variables socioeconómicas, tanto a nivel nacional como entre países.

**Resultados:**

Se analizaron los datos de 105 162 hogares en el año 2000 y 144 018 en el año 2010. Entre el 2000 y 2010, la cobertura doméstica de sal suficientemente yodada aumentó un 6,1% (de un 46,3% a un 52,4%) de media, pero con diferencias regionales: la cobertura cayó un 13,0% (de un 77,5% a un 64,5%) en la República Centroafricana, pero mejoró un 40,4% (de un 22,2% a un 62,6%) en Sierra Leona. Las mejoras de la cobertura fueron mayores en zonas rurales y entre los hogares más pobres, pero las disparidades socioeconómicas nacionales se mantuvieron. Había una pequeña relación entre los cambios de la yodación de la sal y los indicadores socioeconómicos nacionales.

**Conclusión:**

En general, la cobertura de sal doméstica suficientemente yodada ha aumentado durante la última década. No obstante, los cambios son muy diferentes entre países. El objetivo de la yodación universal de la sal sigue siendo lejano en muchos países y requiere de nuevos esfuerzos por parte de los gobiernos, los organismos bilaterales y multilaterales y la sociedad civil.

## ملخص

**الغرض:**

وصف التغييرات في مدى تيسر سبل حصول الأُسر على الملح المعزز باليود فيما يتعلق بالعوامل الاجتماعية والاقتصادية.

**الطريقة:**

قمنا باستخلاص البيانات الخاصة بالملح المعزز باليود المتداول في المنازل من واقع المسوحات العنقودية متعددة المؤشرات التي أُجريت بين عاميّ 2000 و2010. وفي إطار هذه المسوحات، فقد تم اختبار عينات الملح المتداول في المنازل للكشف عن درجة المعالجة باليود باستخدام أدوات موحدة لإجراء الاختبارات السريعة، التي تعطي نتائج لبيان ما إذا كان الملح لم يخضع للمعالجة باليود، أو تمت معالجته على نحو غير كافٍ (أي يحتوي على أقل من 15 **  **جزءًا في **  **المليون)، أو تمت معالجته على نحو كافٍ (بحيث يحتوي على أكثر من 15 **  **جزءًا في **  **المليون). وقمنا باحتساب مؤشرات معالجة الملح المتداول في المنازل باليود بين عاميّ 2000 و2010، مع مراعاة أحجام العينات الواردة في المسح. وقمنا باستكشاف الارتباطات بين هذه المؤشرات والمتغيرات الاجتماعية والاقتصادية، داخل البلدان المختلفة وفيما بينها.

**النتائج:**

قمنا بتحليل البيانات الصادرة عن 105 162 **  **أسرة في عام 2000، و144 018 **  **أسرة في عام **  **2010. وفي الفترة ما بين عامي 2000 و2010، ارتفعت نسبة حصول الأُسر على الملح المعزز باليود بنسبة 6.1% (من 46.3% إلى **  **52.4%) في المتوسط، ولكن مع اختلافات إقليمية: وانخفضت نسبة التغطية إلى **  **13% (من **  **77.5% إلى **  **64.5%) في جمهورية أفريقيا الوسطى ولكن تحسنت النسبة إلى **  **40.4% (من **  **22.2% إلى **  **62.6%) في سيراليون. كانت نسب التحسن في نسبة التغطية أعلى في المناطق الريفية وبين أفقر الأسر، ولكن كانت الفوارق الاجتماعية والاقتصادية داخل الدولة لا تزال قائمة. كانت هناك ارتباطات ضعيفة بين التغييرات في معالجة الملح باليود والمؤشرات الاجتماعية والاقتصادية على المستوى الوطني.

**الاستنتاج:**

بشكل إجمالي، زادت نسبة الحصول على الملح المعزز باليود المتداول في المنازل بشكل كافٍ على مدار العقد الماضي. ومع ذلك، فقد تباينت التغييرات بشكل كبير بين الدول. ولا يزال الهدف من معالجة الملح باليود بعيد المنال بالنسبة للعديد من البلدان ويتطلب بذل جهود جديدة من جانب الحكومات، والوكالات الثنائية ومتعددة الأطراف، والمجتمع المدني.

## 摘要

**目的:**

旨在说明家用碘盐普及情况的变化与社会经济因素的相关性。

**方法:**

我们从 2000 年至 2010 年进行的多指标类集调查中提取了家庭摄取碘盐的数据。作为调查的一部分，通过标准化的快速检测试剂盒检测家用盐，验证是否为碘盐、碘含量不足，少于 15 ppm 或充分加碘（超过 15 ppm）。 考虑到调查抽样权重，我们计算了 2000 年至 2010 年家庭食盐加碘指数。 我们研究了在国家内部和国家之间这些指标与社会经济变量的相关性。

**结果:**

我们分析了从 2000 年的 105 162 户家庭到 2010 年的 144 018 户家庭的调查数据。在 2000 年至 2010 之间，充分加碘盐的覆盖率上升了 6.1%（从 46.3% 到 52.4%），但是仍有地区差异：中非共和国充分含碘盐的覆盖率下降了 13.0％（从 77.5％ 到 64.5％），塞拉利昂的覆盖率却上升了 40.4％（从 22.2％ 上升到 62.6％）。农村地区和最贫困家庭充分含碘盐覆盖率提高了，但是国家内部的社会经济差距依然存在。 加碘盐的变化与国家社会经济指标之间的关联性不强。

**结论:**

总而言之，过去十年充分含碘的家用盐覆盖率上升了。 然而，不同国家之间变化的差异很大。 对于许多国家而言，全民食盐加碘的目标仍然遥不可及，需要政府、双边和多边机构以及民间社团作出新的努力。

## Резюме

**Цель:**

Описать изменения, произошедшие в семьях в отношении доступа к иодированной соли в связи с социально-экономическими факторами.

**Методы:**

Данные по потреблению иодированной соли семействами были взяты из многоиндикаторных кластерных обследований, проведенных в 2000 и 2010 годах. В рамках этих обследований образцы соли, употребляемой в домашнем хозяйстве, проверялись на содержание иода с помощью стандартных наборов для экспресс-тестирования, которые показывают, является ли соль неиодированной, недостаточно иодированной (менее 15 частей на миллион) или достаточно иодированной (более 15 частей на миллион). С учетом весовых коэффициентов пробы были рассчитаны показатели йодирования домашней соли в 2000 и 2010 годах. Были изучены связи между этими показателями и социально-экономическими переменными как внутри стран, так и между странами.

**Результаты:**

Нами были проанализированы данные для 105 162 семейств в 2000 году и для 144 018 семейств в 2010 году. В период между 2000 и 2010 годами доля семейств, пользовавшихся достаточно иодированной солью, выросла в среднем на 6,1% (с 46,3 до 52,4%), но при этом были выявлены региональные отличия: доля таких семейств упала на 13% в Центральноафриканской Республике (с 77,5 до 64,5%), но выросла на 40,4% (с 22,2 до 62,6%) в Сьерра-Леоне. Улучшение было заметнее в сельской местности и среди самых бедных семейств, но при этом сохранялось неравенство социально-экономических показателей внутри страны. Между социально-экономическими показателями на национальном уровне и изменениями в потреблении йодированной соли была выявлена слабая взаимосвязь.

**Вывод:**

В целом доля семейств, использующих достаточно иодированную соль, за последнее десятилетие выросла. Однако от страны к стране изменения значительно варьировались. Многие страны все еще далеки от достижения цели всеобщего обеспечения иодированной солью, и для этого требуется возобновление работы со стороны правительств, двусторонних и многосторонних учреждений и гражданского общества.

## Introduction

The International Council for Control of Iodine Deficiency Disorders (ICCIDD) estimated recently that 28.5% of the world’s population have insufficient dietary iodine intake as indicated by a urinary iodine concentration less than 100 µg/L.^1^ Proportions of the population with iodine deficiency are higher in countries in Africa, South-east Asia, the Eastern Mediterranean regions and eastern Europe than in other parts of the world. Among adults, iodine deficiency leads to an enlarged thyroid gland (goitre). Maternal iodine deficiency during pregnancy can cause stillbirth or mental and physical growth deficits among children.^2^^,^^3^

Since 1994, universal salt iodization has been recommended by the World Health Organization (WHO) and the United Nations Children’s Fund (UNICEF) as a safe and cost-effective strategy to ensure sufficient dietary iodine intake.^4^ The advantages of using salt as a vehicle of the delivery of iodine to people are as follows: (i) salt is one of the few commodities consumed daily by everyone regardless of geography and culture; (ii) the numbers of salt producers are usually limited in each country, allowing effective monitoring of the quality of salt iodization; (iii) iodization is a well-established method that is relatively easy to transfer and implement at a reasonable cost; and (iv) consumer acceptance is high because iodization does not affect the colour, taste or odour of salt. A recent meta-analysis of 87 studies worldwide showed that iodized salt reduces goitre with a pooled relative risk of 0.30 (95% confidence interval, CI: 0.23–0.41) and cretinism with a pooled odds ratio 0.13 (95% CI: 0.08–0.20).^5^ Intelligence quotient (IQ) scores increased by an average of 8.18 points, (95% CI: 6.71–9.65) and urinary iodine concentration by an average of 59.22 µg/L, (95% CI: 50.40–68.04) among children.^5^

Most countries passed salt iodization legislation and introduced salt iodization and iodine deficiency disorders’ control programmes to ensure that more than 90% of households have access to adequately iodized salt, containing 15–40 parts per million (ppm) of iodine.^6^ However, not all salt iodization laws comply fully with the universal salt iodization strategy.^4^ In 2013, for example, only 22 of 25 countries in south and east Asia and the Pacific had salt iodization legislation and only 11 had compulsory iodization of salt for use in food processing industries and households.^7^ Many countries, including Brunei Darussalam, Indonesia, Myanmar, Ukraine and Viet Nam still permit production and sale of non-iodized salt.

Global household coverage of iodized salt increased dramatically during the 1990s from less than 10% to 66%.^8^ In 2011, approximately 70% of all households globally had access to adequately iodized salt.^9^^,^^10^ Among 128 countries with available data on iodized salt, household coverage is greater than 90% in 37 countries, 50–90% in 52 countries and less than 50% in 39 countries.^11^ Countries with the least access to iodized salt are in Africa, the eastern Mediterranean and south-east Asia regions.^11^

Household coverage with iodized salt is a key indicator in Multiple Indicator Cluster Surveys (MICS), which are international household surveys initiated by UNICEF. Here we use MICS data to describe changes in household coverage with iodized salt between 2000 and 2010. We also describe patterns of coverage in relation to socioeconomic factors, within and between countries.

## Methods

These surveys involve a nationally representative sample of between 5000 and 40 000 households using a multistage, cluster sampling technique. Data are collected through home visits and structured face-to-face interviews by national data collection teams; household salt samples are tested for iodine content by the interviewer using standardized test kits. The kits contain a starch-based solution that turns blue if iodine is present. The intensity of the colour varies with the amount of iodine and by matching it with the colour chart the iodine concentration can be ascertained. Salt containing 15 ppm or more of iodine is considered to be adequately iodized in MICS. Results are categorized as follows: (i) not iodized; (ii) iodized at more than 0 ppm and less than 15 ppm; (iii) iodized at 15 ppm or more; (iv) no salt in the household; and (v) not tested.

An index of household wealth is constructed on the basis of household characteristics, including the main materials of the dwelling’s floor, roof and exterior walls; main type(s) of fuel used for cooking; source of drinking water; type of sanitation facility; and household assets.

### National indicators

The country-level socioeconomic indicators used in this study are gross domestic product per capita (GDP) and the Human Development Index (HDI) – a composite index reflecting life expectancy, education and the proportion of the population living above the international poverty line income.^12^ HDI ranges from 0 (the worst) to 1 (the best). Country HDIs are reported annually in Human Development Reports from the United Nations Development Programme. Estimates of GDP per capita are provided annually by the World Bank.^13^ MICS data from rounds two (in 2000), three (in 2005) and four (in 2010) were downloaded from the MICS website.^14^ We analysed data for the 11 countries with data on household coverage with iodized salt in rounds two and four.

### Analysis

Indices of household salt iodization in 2000 and 2010 were calculated, taking into account the sampling weights in each survey. We calculated two indices of the proportion of households with adequately iodized salt: (i) the number of households with salt iodized to at least 15 ppm divided by the total number of households surveyed; and (ii) the proportion of households with adequately iodized salt among households with any iodized salt. These indices were also calculated for urban and rural households and by quintiles of the household wealth index.

We calculated the median proportion of households with adequately iodized salt for the year 2000 and 2010 by national HDI and GDP. Kendall's tau correlation coefficients were calculated to measure the associations among indices.

## Results

The total number of households included in the analyses ranged from 3801 to 24 448 in 2000 and from 7736 to 35 635 in 2010 (Table 1). The exclusion rates (missing data or salt not tested) ranged from 0.05% in the Republic of Moldova to 9.28% in Swaziland in 2000 (overall 2.15%), and from 0.18% in Iraq to 5.66% in Chad in 2010 (overall 2.13%).

**Table 1:** Number of households included in the study on access to iodized salt in 11 low- and lower-middle-income countries, 2000 and 2010

WHO region, country	No. of households	
	Year 2000	Year 2010
**African**		
Central African Republic	13 555	11 429
Chad	5 277	15 458
Democratic Republic of the Congo	8 436	11 317
Kenya	8 883	7 736
Sierra Leone	3 812	11 192
Swaziland	3 801	4 717
**European**		
Republic of Moldova	10 375	10 719
**Eastern Mediterranean**		
Iraq	12 990	35 635
Sudan	24 448	14 644
**Western Pacific **		
Mongolia	5 972	9 615
Viet Nam	7 613	11 556
**Total**	**105** **162**	**144** **018**

### Indicators

#### All households

**Fig. 1:**
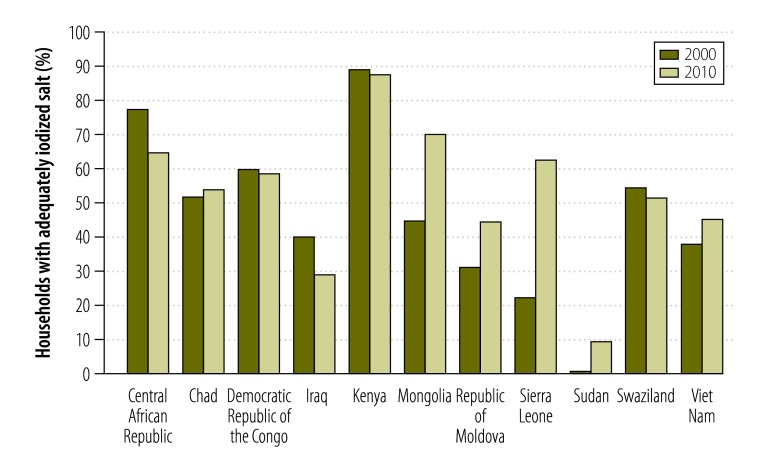
Proportion of households with adequately iodized salt by country, 2000 and 2010

The proportion of households with adequately iodized salt varied widely among the 11 countries (Fig. 1). Between 2000 and 2010, household coverage of adequately iodized salt increased by 6.1% on average, but with regional differences: coverage fell by 13.0% in the Central African Republic but improved by 40.4% in Sierra Leone (Table 2).

**Table 2:** Proportion of households with adequately iodized salt among all households survey, 11 countries, 2000 and 2010

Country	Proportion of households with adequately iodized salt, % 2000; % 2010 (difference)							
	Area			Household wealth index				
	Urban	Rural		Quintile 1 (poorest)	Quintile 2	Quintile 3	Quintile 4	Quintile 5 (richest)
Central African Republic	78; 69.3 (−8.7)	77.3; 62 (−15.3)		72.6; 55.8 (−16.8)	77.6; 62.4 (−15.2)	78.2; 65.2 (−13)	79.3; 70.1 (−9.2)	82.6; 74.7 (−7.9)
Chad	59.4; 59.4 (0.0)	49.4; 52.1 (2.7)		49.1; 43 (−6.1)	45.1; 52.2 (7.1)	47.1; 53.2 (6.1)	56.3; 59.3 (3.0)	60.6; 63.7 (3.1)
Democratic Republic of the Congo	61.8; 58.6 (−3.2)	59; 58.6 (−0.4)		56.4; 57.5 (1.1)	56.9; 57.2 (0.3)	61.3; 57.4 (−3.9)	58.5; 56.3 (−2.2)	67.1; 65.6 (−1.5)
Iraq	42.6; 33.7 (−8.9)	33.4; 15.8 (−17.6)		30.1; 14.9 (−15.2)	55.2; 22.3 (−32.9)	45.8; 26.9 (−18.9)	24; 32.5 (8.5)	44.8; 47.4 (2.6)
Kenya	86.5; 89.5 (3.0)	89.7; 87.4 (−2.3)		89.3; 83.7 (−5.6)	90.4; 86.3 (−4.1)	88.3; 87.5 (−0.8)	88.3; 90.1 (1.8)	88.3; 89.2 (0.9)
Mongolia	62.4; 73.9 (11.5)	28.3; 55.6 (27.3)		21.9; 52.7 (30.8)	29.7; 67.8 (38.1)	44.0; 74.7 (30.7)	56.8; 78.9 (22.1)	69.2; 76.0 (6.8)
Republic of Moldova	36.3; 61.3 (25.0)	27.5; 34 (6.5)		27.2; 23.5 (−3.7)	28.6; 35.9 (7.3)	28.3; 46.2 (17.9)	32.3; 56.6 (24.3)	39.5; 68.0 (28.5)
Sierra Leone	26.0; 63.4 (37.4)	20.6; 62.3 (41.7)		15.8; 56.6 (40.8)	16.6; 62.6 (46)	22.4; 63.8 (41.4)	27.0; 64.3 (37.3)	29.2; 66.5 (37.3)
Sudan	0.8; 10.4 (9.6)	0.2; 8.9 (8.7)		0.2; 18.5 (18.3)	0.3; 9.4 (9.1)	0.4; 6.4 (6.0)	0.8; 5.0 (4.2)	0.8; 6.4 (5.6)
Swaziland	59.7; 57.4 (−2.3)	51.7; 48.5 (−3.2)		50.1; 39.9 (−10.2)	52.8; 48.7 (−4.1)	58.0; 50.4 (−7.6)	55.0; 49.8 (−5.2)	56.5; 63.3 (6.8)
Viet Nam	50.4; 44.4 (−6.0)	33.9; 45.4 (11.5)		30.5; 47.6 (17.1)	31.5; 40.5 (9.0)	33.8; 44.0 (10.2)	40.3; 47.4 (7.1)	54.0; 46.3 (−7.7)
Median of the country proportions	54.8; 59.0 (4.2)	33.9; 50.3 (16.4)		30.3; 45.3 (15.0)	38.3; 50.5 (12.2)	44.9; 51.8 (6.9)	47.7; 56.4 (8.7)	55.3; 64.6 (9.3)

Coverage was generally higher in urban than in rural areas. Overall, there were greater improvements, but from a lower base, in rural than in urban areas. Coverage was higher in wealthier households; however, the largest improvement in coverage over the 10 years was among the poorest groups (Table 2).

There was a negative correlation between the proportion of households with adequately iodized salt in the year and the change in coverage over 10 years (correlation coefficient = −0.58). There were weak correlations between the changes in coverage from 2000 to 2010 and HDI (correlation coefficient = 0.11) or GDP (correlation coefficient = 0.05). The improvements were slightly higher in the countries with higher HDI and GDP (Table 3). However, coverage was lower in countries where HDI and GDP were high.

**Table 3:** Median proportion of households with adequately iodized salt by socioeconomic factors, 11 countries, 2000 and 2010

Factor	Median proportion		Difference
	2000	2010	
**All 11 countries**	42.4	52.7	10.3
**2010 HDI**			
Low (4 countries, HDI < 0.46)	55.8	56.1	0.3
Medium (7 countries)	40.0	45.1	5.1
**2010 GDP**			
Low-income (5 countries, GDP< US$ 1000)	59.8	62.6	2.8
Lower middle-income (6 countries)	38.1	45.1	7.0

GDP: gross domestic product; HDI: human development index; US$: United States dollars.

#### Households with adequately iodized salt

Among households with iodized salt, coverage with adequately iodized salt increased by 5.1% on average between 2000 and 2010. Coverage improved slightly more in urban (2.6%) than in rural (1.6%) areas (Table 4). The poorest households had the smallest changes. The countries with higher HDI and higher GDP had greater improvements from lower initial levels (Table 5).

**Table 4:** The proportion of households with adequately iodized salt among households with any iodized salt, 11 countries, 2000 and 2010

Country	Proportion of households with adequately iodized salt, % 2000; % 2010 (difference)							
	Area			Household wealth index				
	Urban	Rural		Quintile 1 (poorest)	Quintile 2	Quintile 3	Quintile 4	Quintile 5 (richest)
Central African Republic	89.2; 82.6 (−6.6)	89.8; 84.5 (−5.3)		87.9; 86.0 (−1.9)	91.0; 83.1 (−7.9)	89.0; 84.2 (−4.8)	89.3; 83.1 (−6.2)	90.9; 82.4 (−8.5)
Chad	82.3; 74.1 (−8.2)	79.4; 72.8 (−6.6)		81.1; 68.3 (−12.8)	76.8; 74.3 (−2.5)	78.8; 72.4 (−6.4)	79.3; 73.8 (−5.5)	83.8; 76.3 (−7.5)
Democratic republic of the Congo	82.5; 78.2 (−4.3)	74.7; 80.6 (5.9)		72.6; 79.3 (6.7)	76.1; 78.4 (2.3)	76.8; 80.6 (3.8)	75.0; 79.2 (4.2)	85.5; 82.3 (−3.2)
Iraq	77.6; 55.6 (−22.0)	73.6; 42.3 (−31.3)		72.7; 37.7 (−35.0)	85.3; 45.2 (−40.1)	80.0; 51.5 (−28.5)	62.6; 55.6 (−7.0)	75.5; 65.9 (−9.6)
Kenya	90.0; 98.4 (8.4)	93.9; 98.5 (4.6)		94.3; 98.3 (4.0)	94.8; 98.4 (3.6)	92.4; 98.0 (5.6)	91.9; 98.6 (6.7)	91.3; 98.5 (7.2)
Mongolia	90.7; 87.2 (−3.5)	88.0; 82.7 (−5.3)		90.1; 82.7 (−7.4)	86.6; 85.4 (−1.2)	87.7; 85.5 (−2.2)	90.2; 89.2 (−1.0)	92.1; 87.9 (−4.2)
Republic of Moldova	49.6; 80.6 (31.0)	39.0; 72.3 (33.3)		36.8; 66.1 (29.3)	39.2; 73.1 (33.9)	41.5; 78.6 (37.1)	46.1; 78.4 (32.3)	53.9; 81.3 (27.4)
Sierra Leone	56.6; 79.3 (22.7)	54.3; 82.1 (27.8)		48.9; 80.4 (31.5)	50.7; 81.4 (30.7)	52.2; 84.9 (32.7)	55.0; 80.8 (25.8)	65.2; 78.9 (13.7)
Sudan	56.0; 58.4 (2.4)	51.1; 67.5 (16.4)		61.5; 72.1 (10.6)	56.3; 68.7 (12.4)	54.5; 61.0 (6.5)	64.1; 49 (−15.1)	45.8; 55.0 (9.2)
Swaziland	68.0; 65.9 (−2.1)	61.8; 57.4 (−4.4)		62.3; 51.9 (−10.4)	63.1; 57.7 (−5.4)	65.8; 58.8 (−7.0)	63.4; 58.1 (−5.3)	65.3; 69.6 (4.3)
Viet Nam	73.5; 71.6 (−1.9)	61.5; 75.2 (13.7)		57.3; 73.9 (16.6)	60.3; 71.3 (11.0)	61.8; 75.4 (13.6)	67.6; 76.4 (8.8)	74.2; 73.4 (−0.8)
Median of the country proportions	75.6; 78.2 (2.6)	73.6; 75.2 (1.6)		72.6; 73.9 (1.3)	76.1; 74.3 (−1.8)	76.7; 78.6 (1.9)	67.6; 78.4 (10.8)	75.5; 78.9 (3.4)

**Table 5:** Median proportion of households with adequately iodized salt among households with any iodized salt by socioeconomic factors, 2000 and 2010

Factor	Median proportion		Difference
	2000	2010	
**All 11 countries**	70.8	75.9	5.1
**2010 HDI**			
Lowest (4 countries, HDI < 0.46)	78.6	80.5	1.9
Better-off (7 countries)	65.0	74.1	9.1
**2010 GDP**			
Lowest (5 countries, GDP< US$ 1000)	80.1	81.1	1.0
Better-off (6 countries)	64.0	74.1	10.1

GDP: gross domestic product; HDI: human development index; US$: United States dollars.

## Discussion

We examined the household coverage of adequately iodized salt in 11 countries that have relevant Multiple Indicator Cluster Survey data from both 2000 and 2010. Overall, there has been a remarkable improvement in the proportions of households with adequately iodized salt, but there are substantial inter-country differences. Four countries (Mongolia, the Republic of Moldova, Sierra Leone and Sudan) made improvements, five countries (Chad, the Democratic Republic of the Congo, Kenya, Swaziland and Viet Nam) were relatively stable and two countries (the Central African Republic and Iraq) had reductions in coverage.

The four countries with improvements in coverage all had low initial coverage. In these countries, efforts by national governments, international agencies and the mass media to promote the production and consumption of iodized salt were implemented during this period (Table 6). The Republic of Moldova relies entirely on imports of household salt; in the late 1990s the government released a decree banning the importation of non-iodized salt.^15^ UNICEF supported the National Maternal and Child Health Programme during 2000 to 2004 in advocacy, communication, monitoring, evaluation and legislation for salt iodization. Iodization equipment for one main salt importer was supplied to enable the initiation of domestic iodization in the Republic of Moldova.^20^ In 2002, a situation analysis was conducted in the Republic of Moldova, followed by a 3-month mass media campaign. Two national multi-sector workshops developed a collaborative plan of action to eliminate iodine deficiency. As a result, a National Programme to Eliminate Iodine Deficiency Disorder, promoting the supply of iodized salt, was started in 2004.^15^

**Table 6:** Characteristics of 11 countries included in the study on salt iodization, 2000–2010

Country	Salt iodization legislation^a^	Salt iodization national programme^a^	Human development^b^	Economic status^c^	Conflict/war
Central African Republic	Mandatory since 1994	Started in 1995	Low	Low income	The Central African Republic Bush War (2004–2008)
Chad	Voluntary	Started before 2000	Low	Low income	Chadian Civil War (2005–2010)
Democratic republic of the Congo	Mandatory since 1994	Started in 1993	Low	Low income	The Second Congo War (1998–2003)
Iraq	Mandatory in 1993	A lack of national commitment, no national programme	Medium	Lower-middle-income	Iraq war (2003–2011)
Kenya	Mandatory	Started in the 1970s	Low	Low income	Kenyan crisis (2007–2008)
Mongolia	Non-iodized salt banned since 2003	Started in 1996	Medium	Lower-middle-income	No
Republic of Moldova	Voluntary	Started in 2004	Medium	Lower-middle-income	No
Sierra Leone	Voluntary	Started before 2000	Low	Low income	The Sierra Leone Civil War (1991–2002)
Sudan	Voluntary	Started in 1989	Low	Lower-middle-income	Sudanese civil war (1983–2005)
Swaziland	Mandatory since 1997	Started before 2000	Low	Lower-middle-income	No
Viet Nam	Mandatory since 1999, changed to voluntary in 2005	Implemented from 1995–2005	Medium	Lower-middle-income	No

^a^ Sources: Begin & Codling,^7^ van der Haar et al.,^15^ Mahfouz et al.,^16^ Azizi.^17^

^b^ Based on the human development index obtained from the Human Development Report 2011.^18^

^c^ Data from World Bank World Development Report 2010.^19^

In Mongolia, the first National Programme on Elimination of Iodine Deficiency Disorder, from 1996 to 2001, introduced iodized salt for food consumption.^21^ The second and third stages of this programme were implemented from 2002 to 2010, and included multiple activities to improve use of iodized salt including legislation and public awareness campaigns. The government released national standards for iodized salt (in 2001), legislation (Prevention of Iodine Deficiency Disorder by Salt Iodization, in 2003) and regulations that mandated salt iodization (in 2006).^22^

The two African countries in this group (Sierra Leone and Sudan) all experienced civil wars during the 1990s, which are likely to have affected implementation of programmes and could account for the low prevalence of households consuming iodized salt in 2000. Government commitments combined with financial and technical support from international agencies including WHO, UNICEF, and ICCIDD contributed to significant changes, in particular in Sierra Leone.

Chad, the Democratic Republic of the Congo and Swaziland had approximately 50% coverage in 2000 and this remained unchanged in 2010. In these countries, no significant changes in the policy and government efforts concerning salt iodization were implemented during this period.

Kenya scaled up its universal salt iodization programme and was successful in sustaining coverage for the decade. In Viet Nam, the National Iodine Deficiency Disorder Control Programme, supported by UNICEF and ICCIDD, was implemented between 1995 and 2005 and led to an increase in the coverage of iodized household salt from 25% in 1993 to 94% in 2005.^23^ In 2005, the government declared that iodine deficiency had been eliminated in Viet Nam, changed the policy about salt iodization from mandatory to voluntary, and significantly reduced the budget allocated for iodine deficiency disorder control activities.^24^ As a result, the coverage of iodized household salt in 2010 reversed to almost the same level it had been in 2000.

In the Central African Republic and Iraq, coverage decreased significantly from 2000 to 2010. It is likely that military conflict prevented implementation of public policies and services for the civilian population, since both countries were seriously affected by wars during this period.

There were disparities in access to adequately iodized salt, both between rural and urban areas and the poorest and the richest in 2000 and 2010. In 2000, the coverage in urban areas was 20.9% higher than in rural areas, but the gap reduced to 8.7% in 2010. Similarly, the proportion of households in the richest quintile with adequately iodized salt was 25.0% higher than that of the poorest quintile households in 2000, but this reduced to 19.3% in 2010. Even though the inequalities have been reduced in the past decade, the differences between the poor and the rich and between urban and rural remain substantial in many countries.

We acknowledge that the pooled statistics used in this study are summaries of national data and are not representative of specific populations or resource-constrained countries in general. However, these findings can inform strategies for achieving the global goal of more than 90% of households with adequately iodized salt. First, the largest improvement in the coverage of adequately iodized household salt in the decade 2000–2010 was in the countries that started at very low levels and had buy-in from national governments and support from international donors and other agencies. This group of countries appears to be implementing scaling-up salt iodization programmes effectively. Countries with coverage of 50% or higher, in which salt iodization had been scaled up, appeared to face challenges to make further improvements. Some countries are experiencing significant difficulties, including military conflicts which undermine progress. Second, socioeconomic disparities in access to adequately iodized salt are substantial in many countries, suggesting that equity should be addressed explicitly in salt iodization policies. Finally, countries affected by war require explicit additional support from international agencies to achieve universal salt iodization during and following military conflict.

A combination of sustained commitments from governments, the salt industry, international donors and civil society has resulted in remarkable advances in household salt iodization in the past 20 years.^25^ Countries with significant achievements had an operational, political and regulatory environment including passing legislation mandating iodization of salt, effective monitoring systems, strong partnerships with the salt industry, and strategic advocacy and communication efforts.^9^^,^^26^ Countries which maintained low coverage or experienced reduced coverage appeared to lack a political will to advance iodization programmes, had poorly developed salt industries reliant mostly on small-scale producers or little local salt production, had weak government inspection and enforcement systems, and/or were involved in military conflict which severely limited the country’s capacity to implement health programmes.

In conclusion, changes in iodized household salt coverage from 2000 to 2010 vary widely among countries. The achievement and maintenance of universal salt iodization appears a remote goal for many resource-constrained countries and requires explicit renewed efforts by governments, bilateral and multilateral agencies and civil society to avoid the burden of iodine deficiency disorders in the population.
